# Establishment of a selection marker recycling system for sequential transformation of the plant‐pathogenic fungus *Colletotrichum orbiculare*

**DOI:** 10.1111/mpp.12766

**Published:** 2018-12-05

**Authors:** Naoyoshi Kumakura, Akiko Ueno, Ken Shirasu

**Affiliations:** ^1^ RIKEN Center for Sustainable Resource Science 1‐7‐22 Suehiro‐cho, Tsurumi‐ku Yokohama 230‐0045 Japan; ^2^ Graduate School of Science The University of Tokyo 7‐3‐1, Hongo, Bunkyo‐ku Tokyo 113‐8654 Japan

**Keywords:** *Colletotrichum orbiculare*, Cucurbitaceae plants, plant‐pathogenic fungus, selection marker recycling, *URA3/pyrG*

## Abstract

Genome sequencing of pathogenic fungi has revealed the presence of various effectors that aid pathogen invasion by the manipulation of plant immunity. Effectors are often individually dispensable because of duplication and functional redundancy as a result of the arms race between host plants and pathogens. To study effectors that have functional redundancy, multiple gene disruption is often required. However, the number of selection markers that can be used for gene targeting is limited. Here, we established a marker recycling system that allows the use of the same selection marker in successive transformations in the model fungal pathogen *Colletotrichum orbiculare*, a causal agent of anthracnose disease in plants belonging to the Cucurbitaceae. We identified two *C. orbiculare* homologues of yeast *URA3/pyrG*, designated as *URA3A* and *URA3B*, which can be used as selection markers on medium with no uridine. The gene can then be removed from the genome via homologous recombination when the fungus is grown in the presence of 5‐fluoroorotic acid (5‐FOA), a chemical that is converted into a toxin by URA3 activity. The *ura3a/b *double mutants showed auxotrophy for uridine and insensitivity to 5‐FOA. Using the *ura3a/b* mutants, transformation with the *URA3B *marker and its removal were successfully applied to disrupt the virulence‐related gene, *PKS1*. The *pks1* mutants showed a reduction in virulence, demonstrating that the method can be used to study virulence‐related genes in *C. orbiculare*. The establishment of a *URA3*‐based marker recycling system in plant‐pathogenic fungi enables the genetic analysis of multiple genes that have redundant functions, including effector genes.

## Introduction

Phytopathogens have evolved various strategies to overcome plant immunity, including the use of effectors that facilitate a parasitic lifestyle by regulating their host’s immune system. For example, effectors that target pattern‐triggered immunity, the first layer of plant immunity, have been reported in various pathogens. In turn, plants have developed so‐called resistance proteins to detect effectors, inducing a strong defence response against pathogens, called effector‐triggered immunity. During the process of evolution, phytopathogens and plants have developed mutual attack and defence systems that have resulted in functional redundancy and the duplication of pathogen effectors and plant immunity‐related proteins (Asai and Shirasu, 2015; Hogenhout *et al.*, 2009; Jones and Dangl, 2006).

The *Colletotrichum* genus comprises over 600 species, including hemibiotrophic fungi that cause anthracnose disease in various plants, e.g. economically important crops, vegetables and fruits (Cannon *et al.*, 2012). Therefore, the *Colletotrichum* genus is recognized by researchers in the plant–microbe interaction community as one of the 10 most important phytopathogenic fungi (Dean *et al.*, 2012). Within the genus, *Colletotrichum higginsianum* and *Colletotrichum orbiculare* have been recognized as model pathosystems, as they can infect the model plants *Arabidopsis thaliana *and* Nicotiana benthamiana*, respectively (O’Connell *et al.*, 2004; Perfect *et al.*, 1999; Shen *et al.*, 2001; Takano *et al.*, 2006). The functions of several effectors have been reported in *C. higginsianum *and *C. orbiculare*. For example, the lysin motif domain (LysM) contains effectors ELP1 and ELP2 of *C. higginsianum*, which play dual roles in appressorial function and suppression of chitin‐triggered plant immunity (Takahara *et al.*, 2016). In *C. orbiculare*, the effector NIS1 has a cell death‐inducing effect on *N. benthamiana* in an *SGT1*‐ and *HSP90*‐dependent manner (Yoshino *et al.*, 2012). Furthermore, transcriptome analysis has revealed a characteristic expression pattern of effector‐encoding genes during the infection stage transition of *C. higginsianum* and *C. orbiculare*, implying that the coordinate expression of different sets of effectors is orchestrated for successful infection (Gan *et al.*, 2013; Kleemann *et al.*, 2012; O’Connell *et al.*, 2012). Although the functions of several effectors have been elucidated, the vast majority of *Colletotrichum* effectors are still obscure. One reason for this lack of knowledge is the limited availability of selection markers for transformation. There are only four reported combinations of antibiotics (bialaphos, geneticin/G418, hygromycin and sulfonylurea) and corresponding resistance genes that can be used for the transformation of *C. higginsianum *and *C. orbiculare*, making it difficult to analyse redundant effectors (Chung *et al.*, 2002; Dallery *et al.*, 2017; Irieda *et al.*, 2014).

In general, the marker recycling method is used to resolve the limitation of selection markers (Kopke *et al.*, 2010; Zhang *et al.*, 2017). For example, in *Aspergillus* fungi, the *pyrG* (a homologue of *URA3*)‐based marker recycling system has been used and developed (d’Enfert, 1996; Nielsen *et al.*, 2006; Oakley *et al.*, 1987). The *URA3*/*pyrG* gene encodes an orotidine‐5′‐phosphate decarboxylase involved in uridine/uracil synthesis (Weld *et al.*, 2006). In *Saccharomyces cerevisiae*, mutants of *URA3* show growth defects on medium lacking uridine. The uridine auxotrophy of *ura3* enables *URA3* expression cassettes to work as a selection marker for transformation in the *ura3* mutant background. In addition, *URA3/pyrG* can be applied to negative selection (Boeke *et al.*, 1984). Orotidine‐5′‐phosphate decarboxylases encoded by *pyrG* or *URA3* orthologues convert 5‐fluoroorotic acid (5‐FOA), an analogue of the uracil precursor, to 5‐fluorouracil, a toxic compound that inhibits DNA and RNA synthesis (Flynn and Reece, 1999). Therefore, when *URA3/pyrG* is positioned between homologous sequences, excision of the genomic *URA3/pyrG* sequence by homologous recombination can be selected by 5‐FOA treatment. By utilizing this strategy, the *URA3/pyrG* expression cassette can be removed from the fungal genome. Indeed, the *URA3/pyrG*‐based marker recycling system has been utilized in *S. cerevisiae *(Alani *et al.*, 1987), *Aspergillus nidulans *(Nielsen *et al.*, 2006; Oakley *et al.*, 1987), *Aspergillus fumigatus *(d’Enfert, 1996), *Neurospora crassa* (Turner *et al.*, 1997)*, Candida albicans* (Fonzi and Irwin, 1993) and *Mucor circinelloides* (Garcia *et al.*, 2017). However, this system has never been applied to phytopathogenic fungi.

Here, we report the establishment of a *URA3*‐based marker recycling method in *C. orbiculare* 104‐T. As a proof of concept, we knocked out *PKS1*, a gene encoding a polyketide synthase that is required for melanin synthesis involved in virulence (Takano *et al.*, 1995), using the *URA3B* (one of two *pyrG* homologues in *C. orbiculare*) expression cassette as a selection marker. The *PKS1* mutants showed reduced virulence on plants, consistent with previous studies demonstrating that the *URA3B* selection marker can be applied to study virulence‐related genes. In the *pks1* mutant background, *DMAT3*, a secondary metabolism key enzyme encoding gene, was disrupted using the *URA3B* selection marker, demonstrating that the marker recycling system can be applied to sequential transformation and gene deletion. The establishment of a *URA3*‐based marker recycling system enables genes that have redundant functions, such as effectors in pathogenic fungi, to be studied.

## Results

### The *C. orbiculare *genome encodes two *URA3/pyrG *homologues, *URA3A *and *URA3B*

**Figure 1:**
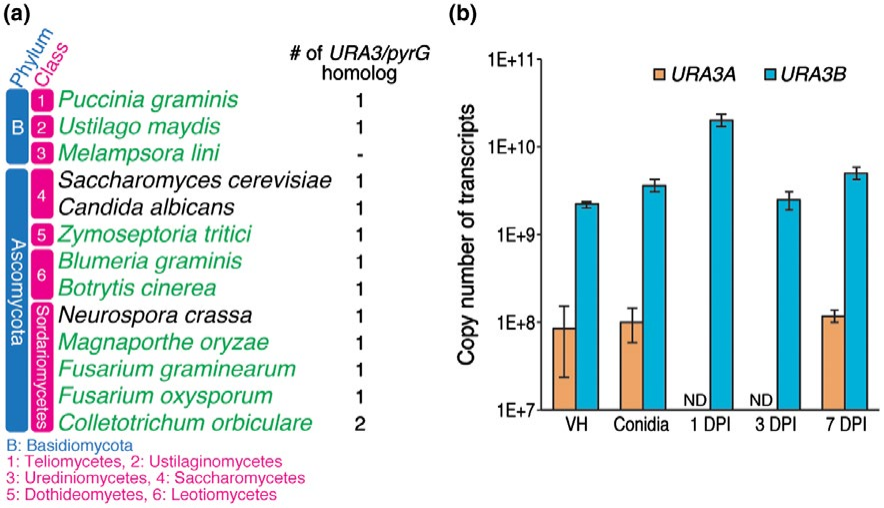
*URA3/pyrG* homologues are conserved in plant fungal pathogens. (a) The number of predicted *URA3*/*pyrG* homologues in selected plant fungal pathogens (green type) and model fungi (black type). Homologues were predicted by BLAST search using the *Saccharomyces cerevisiae* Ura3p amino acid sequence as a query. (b) *URA3A* and *URA3B* expression profiles quantified by reverse transcription‐quantitative polymerase chain reaction (RT‐qPCR) analysis. Bars represent the absolute number of transcripts. RNAs were extracted from vegetative hyphae (VH), conidia, epidermal cells at 1 day post‐inoculation (DPI), epidermal cells at 3 DPI and whole leaf tissue at 7 DPI. Total RNA levels were normalized using the *RIBOSOMAL PROTEIN I5* gene (Cob_11000), as reported previously (Gan et al., 2013). Error bars represent standard errors. *n* = 3. ND indicates not detected. Primers used to detect *URA3A*, *URA3B* and *RPI5* are listed in Table S3 (see Supporting Information). [Colour figure can be viewed at wileyonlinelibrary.com]

To check whether URA3 homologues are present in plant‐pathogenic fungi, including *C. orbiculare*, BLAST (blastp; default setting) search was performed using *S. cerevisiae* Ura3p as a query. Figure 1a shows that each of the 10 different plant fungal pathogens, which were selected as the 10 most important fungal pathogens based on scientific/economic importance (Dean *et al.*, 2012), has at least one *URA3/pyrG* homologue, except for *Melampsora lini.* In particular, *C. orbiculare* has two putative *URA3/pyrG* genes, named *URA3A *(Cob_06825) and *URA3B *(Cob_03887). As shown in Fig. S1 (see Supporting Information), URA3A is more similar than URA3B to *S. cerevisiae *Ura3p*. *However, reverse transcription‐quantitative polymerase chain reaction (RT‐qPCR) analysis revealed that *URA3B* is the major *URA3 *gene, because of its constitutive expression in all tested developmental stages (Fig. 1b)*. *This notion is supported by the fact that *Fusarium oxysporum* URA3, which is the only predicted URA3 homologue in the genome, is more similar to URA3B than URA3A (Fig. S1).

### *URA3A *and *URA3B* double knock‐out mutants exhibit uridine auxotrophy and 5‐FOA insensitivity

**Figure 2:**
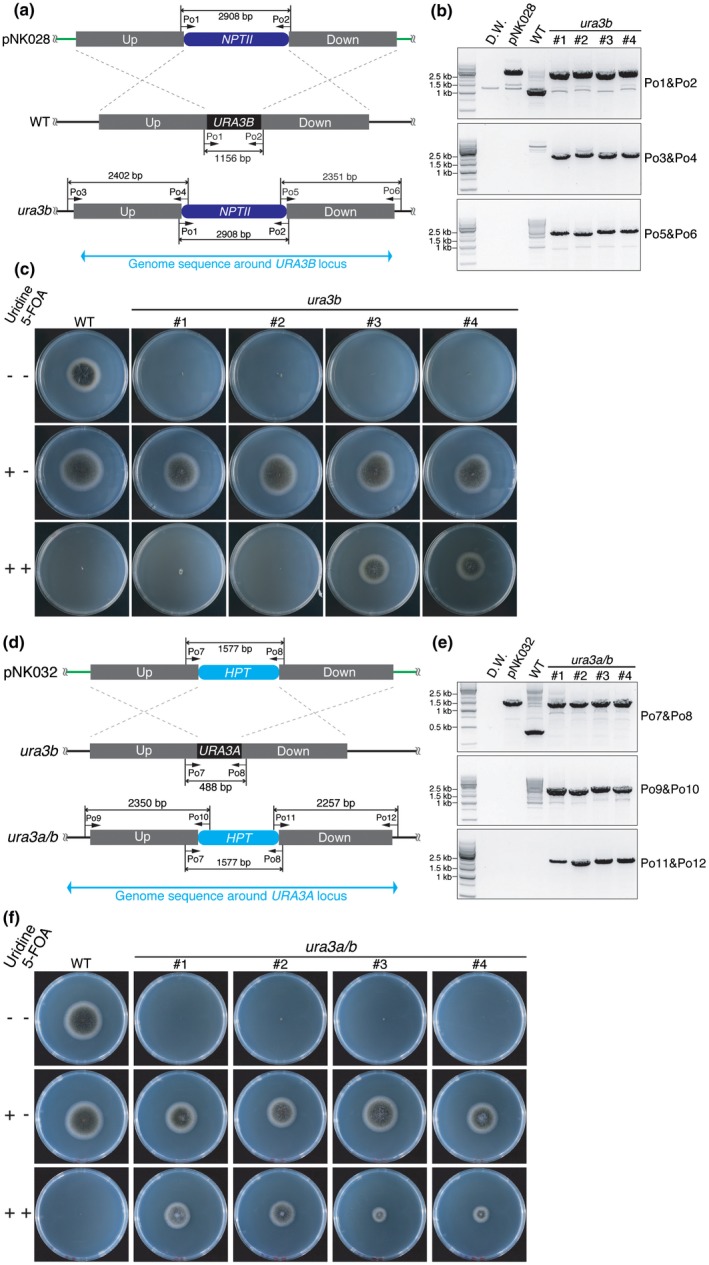
*URA3A* and *URA3B* double knockout mutants show uridine auxotrophy and 5‐fluoroorotic acid (5‐FOA) insensitivity. (a) Schematic diagrams of *URA3B* (Cob_03887) knockout in *Colletotrichum orbiculare* 104‐T wild‐type (WT) strain. The pNK028 plasmid contains 2 kb of upstream (Up) and downstream (Down) sequences of the *URA3B* coding sequence (CDS). The *neomycin phosphotransferase II* (*NPTII*) expression cassette is located between the Up and Down sequences as a selection marker against G418. Black arrows represent primers used for genomic DNA polymerase chain reaction (PCR). (b) Genomic DNA PCR showed that *URA3B* was knocked out. The primer set Po1/Po2 generates 2908‐ and 1156‐bp amplicons from the genome of WT and *ura3b*, respectively. The primer sets Po3/Po4 and Po5/Po6 generate 2402‐ and 2351‐bp bands from the genome of *ura3b*, but not from that of WT*.* (c) WT and four independent *ura3b* strains were cultured on potato dextrose agar (PDA), PDA with 10 mm uridine and PDA with 10 mm uridine and 1 mg/mL 5‐FOA for 6 days at 25 °C in the dark. (d) Schematic diagrams of *URA3A* (Cob_06825) knockout in *ura3b*. The pNK032 plasmid includes the 2‐kb upstream (Up) and downstream (Down) sequences of the *URA3A* locus. The Down sequence partially includes the 3′‐end of the *URA3A *CDS. The *hygromycin phosphotransferase* (*HPT*) expression cassette is located between the Up and Down sequences as a selection marker against hygromycin. (e) Genomic DNA PCR showed that *URA3A* was knocked out, resulting in *ura3a/b* double mutants. *ura3a/b*#1‐2 and *ura3a/b*#3‐4 are descendants of *ura3b*#3 and *ura3b*#2, respectively. The primer set Po7/Po8 generates 488‐ and 1577‐bp amplicons from the genome of WT (or *ura3b*) and *ura3a/b*, respectively. The primer sets Po9/Po10 and Po11/Po12 generate 2350‐ and 2257‐bp bands from the genome of* ura3a/b*, but not from that of WT (or *ura3b*). The primers used are listed in Table S3 (see Supporting Information). (f) The same experiment as described in (c) was performed. Details of each strain are listed in Table S1 (see Supporting Information). [Colour figure can be viewed at wileyonlinelibrary.com]

Because *URA3B* is constitutively expressed, we first knocked out *URA3B* by homologous recombination using the pNK028 plasmid harbouring *neomycin phosphotransferase II* (*NPTII*), a geneticin/G418 resistance gene (Fig. 2a). At least four *ura3b *knock‐out strains were obtained and the gene disruption was confirmed by genomic PCR using the primer sets Po1/Po2, Po3/Po4 and Po5/Po6 (Fig. 2a,b). As predicted, all *ura3b* mutants showed no grow on potato dextrose agar (PDA), but were able to grow on PDA supplemented with 10 mm uridine, suggesting that URA3B is indispensable for uridine synthesis *in vivo* (Fig. 2c). The growth of wild‐type *C. orbiculare* was inhibited by the addition of 1 mg/mL 5‐FOA to PDA, implying that URA3B also synthesizes a toxic compound from 5‐FOA. However, the 5‐FOA sensitivity varied among the *ura3b* mutants (Fig. 2c), suggesting that residual URA3 enzyme activity was contributed by the other URA3 homologue URA3A. Therefore, we decided to knock out *URA3A* in *ura3b* and established four double knock‐out lines by homologous recombination using pNK032 (Fig. 2d)*.* As shown in Fig. 2e, genomic PCR confirmed that the *URA3A* gene was disrupted in *ura3a/b*#1‐4*.* Importantly, all four strains showed uridine auxotrophy and 5‐FOA insensitivity, demonstrating that the URA3‐based enzyme activity in *C. orbiculare* were completely lost in the *ura3a/b*#1‐4 mutants (Fig. 2f).

### The *URA3B *expression cassette functions as a selection marker in *ura3a/b* mutants

**Figure 3:**
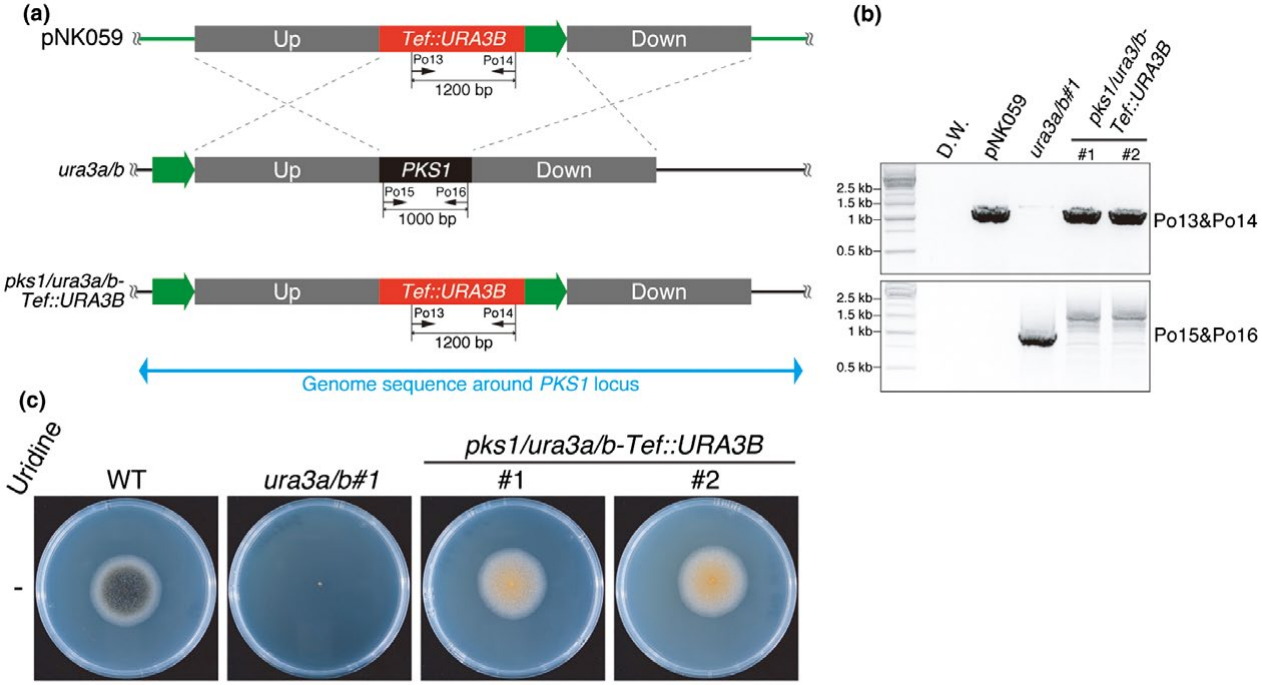
The *URA3B* expression cassette driven by the *Tef* (*TRANSLATION ELONGATION FACTOR*) promoter works as a uridine auxotrophy selection marker in *ura3a/b*. (a) Schematic diagrams of *PKS1* (Cob_09513) knock out in *ura3a/b. *The pNK059 plasmid has 2 kb of upstream (Up) and downstream (Down) sequences of the *PKS1* locus. The Down sequence includes the partial *PKS1 *coding sequence (CDS). The *URA3B* expression cassette driven by the *Tef* constitutive promoter (*Tef::URA3B*) is located between the Up and Down sequences as a uridine auxotrophy marker. Green arrows represent a 500‐bp sequence located upstream of the Up sequence on the *Colletotrichum orbiculare* genome. The 500‐bp sequence is designed to allow homologous recombination induced by 5‐fluoroorotic acid (5‐FOA) treatment for excision of *Tef::URA3B*. (b) Genomic DNA polymerase chain reaction (PCR) showed that *PKS1* was knocked out. The primer set Po13/Po14 generates the 1200‐bp amplicon if the *Tef::URA3B* cassette is present. The primer set Po15/Po16, generates the 1000‐bp band if the *PKS1* CDS is present. The primers used are listed in Table S3 (see Supporting Information). (c) Wild‐type (WT), *ura3a/b#1* and two independent *pks1/ura3a/b‐Tef::URA3B* strains were cultured on potato dextrose agar (PDA) for 6 days at 25 °C in the dark. The details of each strain are listed in Table S1 (see Supporting Information). [Colour figure can be viewed at wileyonlinelibrary.com]

To test whether the uridine auxotrophy selection marker is usable in *C. orbiculare*, *PKS1*, which encodes a polyketide synthase involved in melanin synthesis (Takano *et al.*, 1995, 1997), was targeted for disruption by the pNK059 plasmid. The plasmid has the *URA3B* expression cassette as a selection marker, in which *URA3B* is driven by the *Tef* (*TRANSLATION ELONGATION FACTOR*) promoter of *Aureobasidium pullulans* (Wymelenberg *et al.*, 1997) (Fig. 3a). Successful knock out of *PKS1* was confirmed by genomic PCR (Fig. 3b). Consistently, *pks1/ura3a/b*‐*Tef::URA3B*#1 and *pks1/ura3a/b*‐*Tef::URA3B*#2 showed the albino phenotype, characteristic of *pks1* mutants and caused by a lack of melanin (Takano *et al.*, 1995) (Fig. 3c).

### The excision of the *URA3B *expression cassette can be selected by 5‐FOA treatment, demonstrating establishment of the marker recycling system in *C. orbiculare*

**Figure 4:**
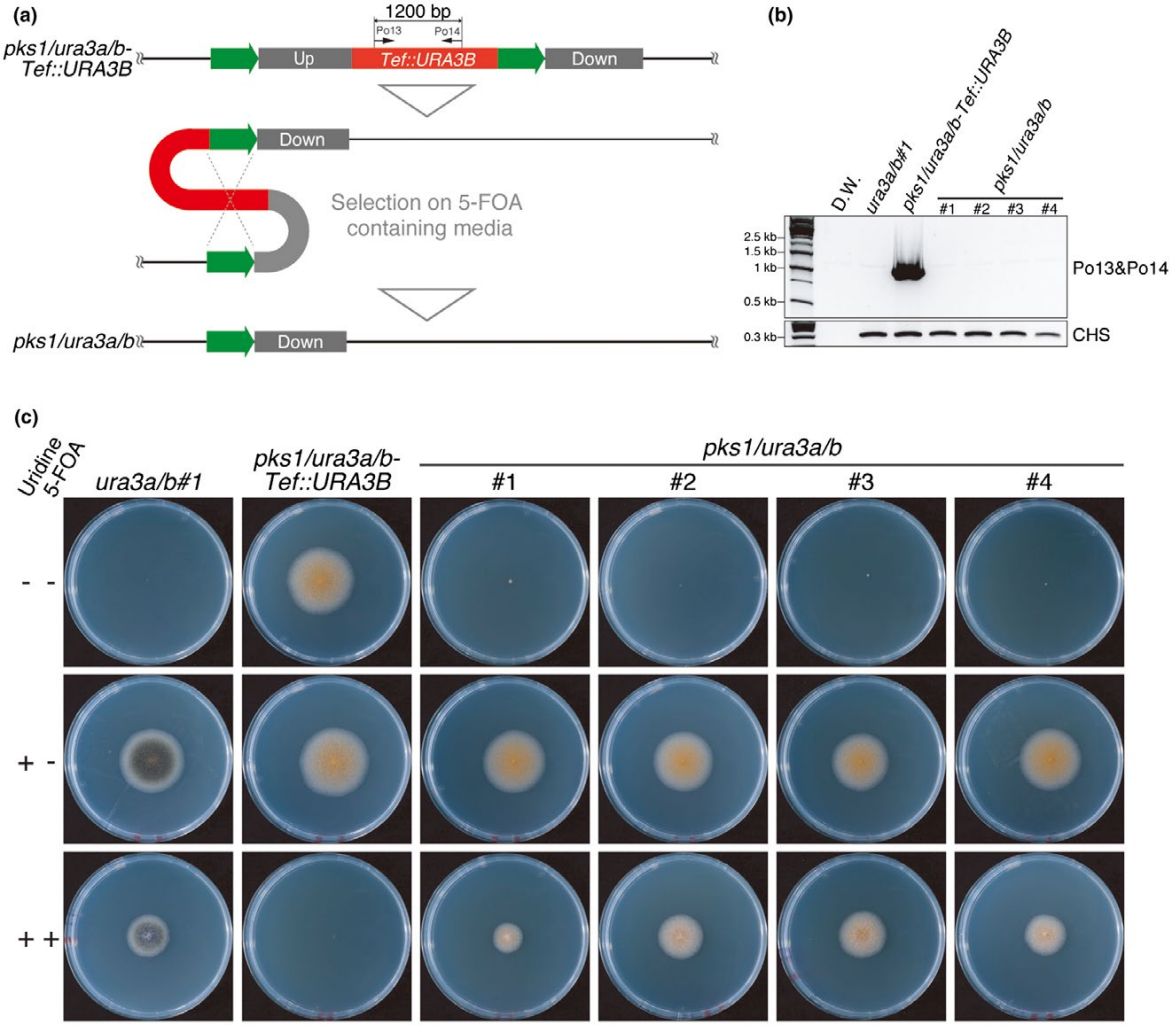
The *Tef::URA3B* cassette was removed by homologous recombination and selected by 5‐fluoroorotic acid (5‐FOA) treatment. (a) Schematic diagrams of the removal of the *Tef::URA3B* cassette (Cob_09513)*. *Green arrows show the homologous 500‐bp sequences for recombination. (b) Genomic DNA polymerase chain reaction (PCR) showed that *Tef::URA3B *was removed. The primer set Po13/Po14 generates the 1200‐bp amplicon if the *Tef::URA3B* cassette is present. The 266‐bp bands corresponding to *CHITIN SYNTHASE *(*CHS*) were amplified using the primer set CHS_79F and CHS_345R (Carbone and Kohn, 1999) to show the presence of genomic DNA. The primers used are listed in Table S3 (see Supporting Information). (c) *ura3a/b*#1, *pks1/ura3a/b‐Tef::URA3B*#1 and *pks1/ura3a/b*#1‐4 strains were cultured on potato dextrose agar (PDA), PDA with 10 mm uridine and PDA with 10 mm uridine plus 1 mg/mL 5‐FOA for 6 days at 25 °C in the dark. All *pks1/ura3a/b*#1‐4 strains showed uridine auxotrophy and 5‐FOA insensitivity similar to *ura3a/b*#1, demonstrating successful removal of the *Tef::URA3B* cassette. The details of each strain are listed in Table S1 (see Supporting Information). [Colour figure can be viewed at wileyonlinelibrary.com]

In the *pks1/ura3a/b*‐*Tef::URA3B* strains, the *Tef::URA3B* cassette is designed to be located between 500 bp of completely homologous sequences (Fig. 4a, green boxes). If *pks1/ura3a/b*‐*Tef::URA3B* is incubated on PDA plates containing 5‐FOA, the strains that lose the *Tef::URA3B* cassette by homologous recombination should be selected. As predicted, *pks1/ura3a/b* strains without the *Tef::URA3B* cassette could be isolated from *pks1/ura3a/b‐Tef::URA3B* strains after growth on PDA containing 1 mg/mL 5‐FOA and 10 mm uridine. Then, using eight randomly selected colonies per strain, the removal of the *Tef::URA3B* cassette was checked by genomic DNA PCR. No bands were observed from genomic DNA of all *pks1/ura3a/b* strains (#1–4 are shown as representatives in Fig. 4b) using the primer set Po13/Po14, which generates 1200‐bp amplicons in the presence of the *Tef::URA3B* cassette. The *pks1/ura3a/b* strains were unable to grow on PDA, but grew on PDA supplemented with 10 mm uridine (Fig. 4c). These strains also show low sensitivity to 5‐FOA treatment, suggesting the absence of the *Tef::URA3B* cassette. Together, we conclude that the removal of the *Tef::URA3B* marker from *pks1/ura3a/b‐Tef::URA3B* can be selected by 5‐FOA treatment.

### *URA3B* marker knock in enables *in planta* virulence assay in *C. orbiculare*

**Figure 5:**
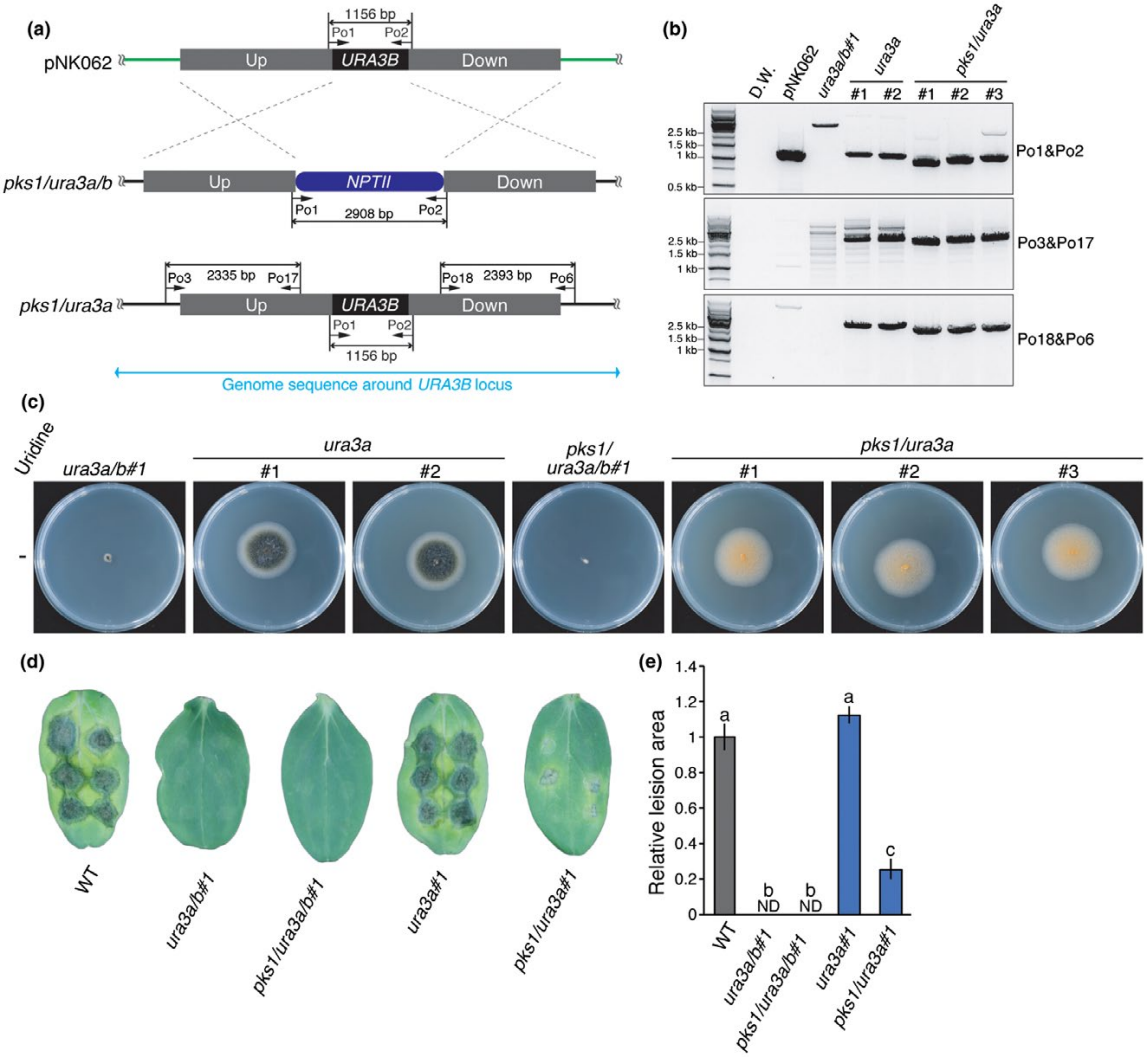
*URA3B* knock‐in to its original locus enabled *in planta* virulence assay using the *ura3a/b* mutant‐based marker recycling system. (a) Schematic diagrams of *URA3B *knock‐in experiments on the *ura3a/b* mutant strains*. *The pNK062 plasmid contains 2 kb of upstream (Up) and downstream (Down) sequences of the *URA3B* coding sequence (CDS) on the *Colletotrichum orbiculare* 104‐T genome. The complete *URA3B *CDS, including intron, is located between the Up and Down sequences. (b) Genomic DNA polymerase chain reaction (PCR) showed that *URA3B* was knocked in, resulting in *ura3a* and *pks1/ura3a *mutants. The primer set Po1/Po2 is described in Fig. 2b. If the *URA3B* CDS is knocked in to *ura3a/b* and *pks1/ura3a/b* mutants, primer sets Po3/Po17 and Po18/Po6 generate 2335‐ and 2393‐bp bands, respectively. The primers used are listed in Table S3 (see Supporting Information). (c) All strains were cultured on potato dextrose agar (PDA) for 6 days at 25 °C in the dark. *ura3a*#1, *ura3a*#2, *pks1/ura3a*#1, *pks1/ura3a*#2 and *pks1/ura3a*#3 strains can grow on PDA without uridine addition, demonstrating the knock‐in of *URA3B*. (d) Conidia of each strain were inoculated onto plants. At 9 days post‐germination, cotyledons of cucumber were inoculated with six drops of 10 µL conidia solution at 5 × 10^5^ conidia/mL. Photographs were taken after 6 days of incubation. The disease symptoms of *ura3a*#1 were similar to those of the WT strain, showing that *URA3B* is able to complement the reduced virulence phenotype of the *ura3a/b* mutant. The reduced virulence phenotype of *pks1/ura3a*#1 reflects the contribution of *PKS1* to the virulence of *C. orbiculare*. The details of each strain are listed in Table S1 (see Supporting Information). (e) The area of each lesion shown in (d) was measured using Image J software. Values were normalized to set WT as unity. *n* = 12. Error bars represent standard errors. ND indicates not detected. Different letters on the bars represent significant differences (Tukey’s test, *P* < 0.01). [Colour figure can be viewed at wileyonlinelibrary.com]

We tested whether the marker recycling system could be applied to study virulence *in planta*. As shown in Fig. 5d, *ura3a/b* mutants did not trigger disease symptoms on cucumber cotyledons, most probably because uridine was not acquired from the host plant. To check whether externally added uridine complements the phenotype of *ura3a/b*, wild‐type, *ura3a/b* and *ura3a/b *supplemented with 10 mm uridine were inoculated onto cucumber cotyledons. Externally added uridine partially complemented the disease phenotype of *ura3a/b* (Fig. S2c,d, see Supporting Information), showing that uridine is required for the virulence of *C. orbiculare*. Then, to determine at which stage the pathogenicity of *ura3a/b *mutants was arrested, the rates of conidial penetration, an early event of infection, were assessed. Although appressoria were formed as in the wild‐type, the *ura3a/b* mutants could not penetrate into the cucumber cells at all (Fig. S2a,b). This deficiency in the penetration rate of *ura3a/b *was partially complemented by externally added uridine (Fig. S2d). These results suggest that infection of *ura3a/b* is arrested at the penetration stage and uridine is required for successful penetration.

Next, we knocked in the *URA3B* gene in *ura3a/b* to its original locus using pNK062 (Fig. 5a). The successful knock in of the *URA3B* gene was confirmed by genomic DNA PCR (Fig. 5b) and phenotypic analysis (Fig. 5c). No significant difference in disease symptoms between wild‐type and *URA3B *knocked‐in strains was observed, demonstrating that *URA3B* alone was sufficient to complement the reduced virulence phenotype of *ura3a/b *(Fig. 5d,e). Then, we knocked in *URA3B* in *pks1/ura3a/b* mutants to produce *pks1/ura3a *mutants, and infected cucumber (*Cucumis sativus*) cotyledons. As shown in Fig. 5d,e, *pks1/ura3a* showed significantly less virulence than *ura3a*, indicating the involvement of *PKS1* in virulence, as described previously (Takano *et al.*, 1995). These data demonstrate that the marker recycling system can be applied to the *in planta* assays to test genes involved in virulence.

### *DMAT3* knock‐out in the *pks1/ura3a/b* mutant using the marker recycling system

**Figure 6:**
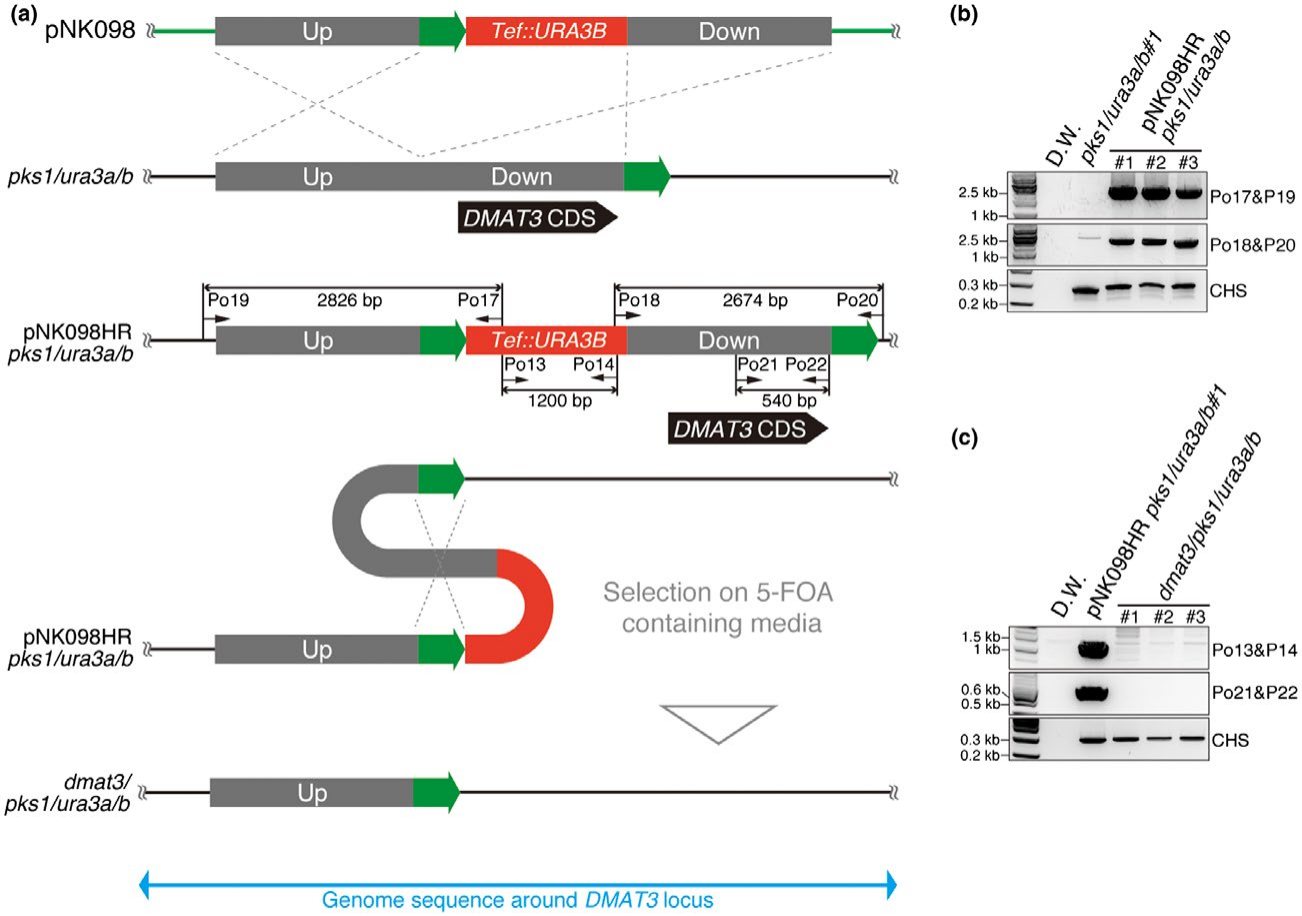
*DMAT3* knock‐out in the *pks1/ura3a/b* mutant. (a) Schematic diagrams of the *DMAT3* coding sequence (CDS) knock‐out experiments in the *pks1/ura3a/b* background. The pNK098 plasmid includes about 2 kb of upstream (Up) and downstream (Down) sequences around the *DMAT3* locus. The Down sequence contains the complete *DMAT3* CDS shown by a black box. Green arrows show homologous 500‐bp sequences for recombination originally from the downstream region of *DMAT3* CDS. (b) Genomic DNA polymerase chain reaction (PCR) showed successful homologous recombination by pNK098, resulting in pNK098HR *pks1/ura3a/b* strains. The primer sets Po17/Po19 and Po18/20 generate 2826‐ and 2674‐bp bands, respectively, from pNK098HR *pks1/ura3a/b*, but not from the genome of *pks1/ura3a/b*. The 266‐bp bands corresponding to *CHITIN SYNTHASE *(*CHS*) were amplified to show the presence of genomic DNA. (c) Genomic DNA PCR showed the successful removal of the *Tef::URA3B* expression cassette and the *DMAT3* CDS. The primer sets Po13/Po14 and Po21/Po22 generate 1200‐ and 540‐bp bands, respectively, from the genome of pNK098HR *pks1/ura3a/b*, but not from that of *dmat3/pks1/ura3a/b*. The primers used are listed in Table S3 (see Supporting Information). [Colour figure can be viewed at wileyonlinelibrary.com]

To check whether the marker recycling system can be repeatedly used for transformation and gene targeting, we performed one round of additional gene disruption in the *pks1/ura3a/b* mutant. As a target, *DMAT3* (Cob_04983), which encodes a predicted secondary metabolite (SM) key gene, dimethylallyl transferase, was selected for gene knock‐out by the pNK098 plasmid (Fig. 6a). First, the *Tef::URA3B* expression cassette was inserted into the upstream region of the *DMAT3 *coding sequence (CDS) in *pks1/ura3a/b*. Successful insertion of the *Tef::URA3B* cassette was confirmed by genomic DNA PCR (Fig. 6b), resulting in three independent strains, named pNK098HR *pks1/uras3/b*. In these strains, the *Tef::URA3B *cassette and the *DMAT3* CDS were located between 500 bp of completely homologous sequences (Fig. 6a, green boxes) for the marker and CDS removal. Second, removal of the *Tef::URA3B* cassette and *DMAT3 CDS* was selected for by incubating pNK098HR *pks1/uras3/b* on PDA plates containing 5‐FOA and uridine. The successful removal of the *Tef::URA3B* cassette and *DMAT3* CDS was confirmed by genomic DNA PCR (Fig. 6c), resulting in three independent *dmat3/pks1/ura3a/b* mutant strains. These results prove the concept of sequential transformation and gene targeting using the marker recycling system reported here.

## Discussion

The limited availability of selection markers represents a bottleneck for effector studies in many plant‐pathogenic fungi. Here, we report the establishment of a marker recycling system using *URA3/pyrG* homologues in the phytopathogenic filamentous fungus, *C. orbiculare*. The *URA3B* cassette was successfully used as a selection marker in *C. orbiculare ura3a/b* double mutants to create *PKS1 *knock‐out lines. Selection for loss of the *URA3B* cassette via homologous recombination can then easily be performed by growth on media supplemented with 5‐FOA. In addition, we successfully knocked out *DMAT3* in the *pks1* mutant background, proving the concept of sequential transformation by the marker recycling system. Importantly, the reintroduction of *URA3B* at its original locus is able to restore the growth of *ura3a/b *mutants to wild‐type levels *in planta*. Thus, using this system, we should be able to assess the function of any gene of interest *in planta*. The avoidance of positional effects is critical, as shown in *Candida albicans*, an opportunistic fungal pathogen of animals, where the virulence phenotype of the fungus in mice varied as a function of the relocation of *URA3* in the genome, as the expression of *URA3* during infection is affected by its genomic locus (Staab and Sundstrom, 2003; Sundstrom *et al*., 2002). The reintroduction of *URA3B* is an additional step in the protocol, but the use of the same vector and the high efficiency of homologous recombination make this process straightforward. As the *URA3B* cassette can be recycled, knock‐out analysis of multiple genes with functional redundancy is now possible in *C. orbiculare*. Similarly, the *URA3*‐based marker recycling system can be applied to other pathogenic fungi that are transformable with a relatively high homologous recombination rate. The genome sequences of other transformable plant pathogens, *Ustilago maydis*, *Zymoseptoria tritici*, *Botrytis cinerea*, *Magnaporthe oryzae*, *Fusarium graminearum*, *F. oxysporum* and *C. higginsianum*, revealed that these organisms contain only one copy of *URA3* (Fig. 1a). Thus, it is possible that setting up the *ura3* knock‐out system in these pathogens may be easier than that in *C. orbiculare*.

The *URA3*‐based marker recycling system offers several advantages over other systems, such as Cre‐*loxP*, Flp‐*FRT* and β‐recombinase‐*six*, which have been used in prokaryotes and eukaryotes (Abuin and Bradley, 1996; Johansson and Hahn‐Hägerdal, 2004; Lambert *et al.*, 2007; Szewczyk *et al.*, 2014; Yuliya *et al.*, 2010; Zhang *et al.*, 2017). Cre, Flp and β‐recombinase catalyse the recombination between sites, named *loxP*, *FRT* and *six*, respectively. As these enzymes lead to the excision of DNA between the two recombination sites, one of the sites is left behind in the genome (Kilby *et al.*, 1993; Wirth *et al.*, 2007). Thus, unlike our homologous recombination‐based *URA3* system, foreign sequences accumulate in the genome if sequential transformation is performed, which potentially induces genome instability. Therefore, the *URA3/pyrG*‐based marker recycling system should allow the creation of much more stable multiple knock‐out lines. Further, the *URA3B* cassette has been demonstrated to function as both a positive and negative selection marker, avoiding the need for opposite markers as used in the β‐recombinase‐based system, which utilizes the bialaphos resistance gene as a positive selection marker for transformation and the thymidine kinase gene as a negative selection marker for excision (Szewczyk *et al.*, 2014). Thus, the *URA3/pyrG*‐based marker recycling system should allow the construction of smaller vectors.

In *Aspergillus nidulans*, a model organism for fungal research, *URA3/pyrG*‐based transformation and related technologies have been developed (Dohn *et al.*, 2018; Oakley *et al.*, 1987). For example, Szewczyk *et al.* (2006) were able to improve the speed of transformation experiments by utilizing PCR fragments (not plasmids) for gene targeting. Further, Nayak *et al.* (2006) identified and knocked out the *A. nidulans* homologue (*nkuA*) of the human *KU70* gene, which is important for non‐homologous end joining of DNA in double‐strand breaks. A lack of *nkuA* reduces the frequency of non‐homologous integration of DNA fragments for transformation, leading to higher gene targeting efficiency. The homologue of *nkuA* has also been knocked out in *C. higginsianum *(Ushimaru *et al.*, 2010), the causal agent of anthracnose on Brassicaceae plants, leading to improved gene targeting efficiency. Thus, it is likely that the method may be applicable to other members of the *Colletotrichum* fungi. The application of these advanced technologies developed in *A. nidulans *and other fungi to *C. orbiculare* is theoretically possible and could make sequential gene targeting and transformation more rapid and easier in combination with the marker recycling system.

In recent years, the genomes of many phytopathogenic fungi, such as *Colletotrichum* species, *M. oryzae*, *U. maydis* and *Z. tritici*, have been sequenced and the presence of multiple effector proteins has been predicted (Dean *et al.*, 2005; Gan *et al.*, 2013; Ma *et al.*, 2010; O’Connell *et al.*, 2012; Spanu *et al.*, 2010). In *Colletotrichum*, transcriptome analysis revealed that the expression of several SM synthesis‐related genes is strongly induced during infection, especially at the biotrophic phase, in addition to effector proteins (Dallery *et al.*, 2017; Gan *et al.*, 2013). These findings suggest that SMs synthesized by fungi may have a virulence function. One SM synthesis‐related gene, *btcAco*, whose expression is induced during infection, was knocked out, and its function was assessed (Gao *et al.*, 2018). Although *btcAco* is involved in SM synthesis, virulence effects in *btcAco* disrupted mutants were not detected (Fig. S3, see Supporting Information). One possible reason for this lack of virulence phenotype is that SMs may also have functional redundancy in virulence. We anticipate that the *URA3/pyrG* marker recycling system will contribute to elucidate the functions of effectors, including proteins, and SMs.

## Experimental Procedures

### Fungal transformation

Fungal transformation was performed using the polyethylene glycol‐mediated protoplast transformation protocol described previously (Kubo *et al.*, 1991). *Colletotrichum orbiculare* 104‐T (MAFF240422) was used as the wild‐type strain (Ishida and Akai, 1969). Derivative strains from *C. orbiculare* 104‐T and plasmids used for transformation are listed in Tables S1 and S2 (see Supporting Information), respectively.

### Selection of removal of the *URA3B* expression cassette after 5‐FOA treatment

The strains *pks1/ura3a/b*‐*Tef::URA3B*#1 (CoNK0031) and *pks1/ura3a/b*‐*Tef::URA3B*#3 (CoNK0033) were cultured on PDA (Nissui Pharmaceutical Co., Ltd., Taito‐ku, Tokyo, Japan) for 6 days and their conidia were collected. About 1 × 10^5^ conidia of CoNK0031 and CoNK0033 were spread onto PDA with 1 mg/mL 5‐FOA monohydrate (Wako Pure Chemical Industries, Ltd., Chuo‐ku, Tokyo, Japan) and 10 mm uridine (Tokyo Chemical Industry Co., Ltd., Chuo‐ku, Tokyo, Japan) in sterilized no. 2 square plates (Eiken Chemical Co., Ltd., Taito‐ku, Tokyo, Japan). Then, the plates were incubated at 25 °C in the dark for 4 days. The surviving colonies were transferred to new PDA with 1 mg/mL 5‐FOA and 10 mm uridine, and incubated under the same conditions for 4 days. The surviving colonies were selected and removal of the *Tef::URA3B* cassette was examined by fungal colony PCR. Selected transformants were designated as *pks1/ura3a/b*#1 (CoNK0041), *pks1/ura3a/b*#2 (CoNK0042), *pks1/ura3a/b*#3 (CoNK0043) and *pks1/ura3a/b*#4 (CoNK0044).

### Plasmid construction

All primers used are listed in Table S3 (see Supporting Information). The genomic DNA of *C. orbiculare* 104‐T used for PCR was isolated as described previously (Gan *et al.*, 2013).

pNK028: PCR‐1 and PCR‐2 were amplified from *C. orbiculare* 104‐T genomic DNA using the primer sets IF‐pII99EcoRV+URA3BUP_F plus IF‐pII99EcoRV+URA3BUP_R and pNK028‐DW_F plus pNK028‐DW_R. The pII99 plasmid harbouring *NPTII*, a geneticin/G418 resistance gene (Namiki *et al.*, 2001), was digested with *Eco*RV (TaKaRa Bio, Inc., Kusatsu, Shiga, Japan) and the larger fragment was fused to PCR‐1 using the In‐Fusion HD Cloning Kit (TaKaRa Bio, Inc.) according to the manufacturer’s instructions, resulting in pNK028P1. pNK028P1 was digested with *Bam*HI (TaKaRa Bio, Inc.) and the larger fragment was assembled with PCR‐2 using the In‐Fusion HD Cloning Kit, resulting in the pNK028 plasmid for the *URA3A* knock‐out harbouring *NPTII*.

pNK032: PCR‐3 and PCR‐4 were amplified from *C. orbiculare* 104‐T genomic DNA using the primer sets URA3AUP_fwd plus URA3AUP_rev and HF‐pNK032‐DW_F plus IF‐pENTR4URA3AKO‐R. PCR‐5 was amplified from the pCB1004 plasmid (Sweigard *et al.*, 1997) using the primer set HygR_fwd plus HF‐pNK032‐HygR_R. PCR‐6 was amplified from pENTR4 Dual Selection (Thermo Fisher Scientific. Inc., Waltham, Massachusetts, USA) using the primer set pENTR4_Dual_selection_F plus pENTR4_Dual_selection_R. PCR‐3 to PCR‐6 were assembled using NEBuilder HiFi DNA Assembly Mix (New England Biolabs, inc., Ipswich, Massachusetts, USA) according to the manufacturer’s protocol, resulting in pNK032 for *URA3B* knock‐out harbouring *hygromycin phosphotransferase* (*HPT*), which confers resistance to hygromycin B.

pNK059: PCR‐7, PCR‐8 and PCR‐9 were amplified from *C. orbiculare* 104‐T genomic DNA using the primer sets IF1‐Cob_09513_UP_F plus HFBK‐pNK059_R, HFBK‐pNK059_F plus Cob_09513_UP(‐500)_R and Cob_09513_Dw_F plus IF3‐Cob_09513KO_DW_R, respectively. PCR‐10, having the *Tef* promoter sequence, and PCR‐11, having the SCD1 terminator, were amplified from Tef‐GFP plasmid using the primer sets HF‐pNK059Frag1_F plus HF‐pNK059Frag1_R and HF‐pNK059Frag3_F plus HF‐pNK059Frag3_R. The sequences of PCR‐10 and PCR‐11 are given in Data S1 (see Supporting Information). PCR‐12 was amplified from *C. orbiculare* 104‐T cDNA using the primer set HF‐pNK059Frag2_F plus HF‐pNK059Frag2_R. PCR‐10 to PCR‐12 were assembled by NEBuilder HiFi DNA Assembly Master Mix, resulting in PCR‐13, which is the *Tef* promoter‐driven *URA3B* expression cassette. PCR‐14 was amplified from the pAGM4723 plasmid (Engler *et al.*, 2014) using pCIH47732_IF_F plus pICH47732_IF_R2. PCR‐7, PCR‐8, PCR‐13, PCR‐9 and PCR‐14 were assembled by NEBuilder HiFi DNA Master Mix, resulting in pNK059.

pNK062: PCR‐15 was amplified from pNK028 using the primer set HF‐pNK062‐BB_F and HF‐pNK062‐BB_R. PCR‐16 was amplified from *C. orbiculare* 104‐T genomic DNA using the primer set HF‐pNK062‐IN_F and HF‐pNK062‐BB_R. PCR‐15 and PCR‐16 were assembled using NEBuilder HiFi DNA Assembly Master Mix, resulting in pNK062.

pNK098: PCR‐17, PCR‐18 and PCR‐19 were amplified from *C. orbiculare* 104‐T genomic DNA using the primer sets HF‐pNK098UP_F plus HF‐pNK098UP_R, HF‐pNK098HS_F plus HF‐pNK098HS_R and HF‐pNK098DOWN_F plus HF‐pNK098DOWN_R, respectively. PCR‐20 harbouring the *URA3B* expression cassette was amplified from pNK059 using the primer set HF‐URA3BCas_F plus HF‐URA3BCas_R. Then, PCR‐6, PCR‐17, PCR‐18, PCR‐19 and PCR‐20 were assembled using NEBuilder HiFi DNA Assembly Master Mix, resulting in pNK098.

### RT‐qPCR

Total RNA isolation and DNA removal were carried out using an RNeasy Plant Mini Kit and RNase‐Free DNase Set (Qiagen, Venlo, Limburg, Netherlands) following the manufacturer’s protocol. cDNAs were synthesized from isolated RNAs with the ReverTraAce qPCR RT Kit (Toyobo Co., Ltd., Kita‐ku, Osaka, Japan) using the included primer mix and following the manufacturer’s instructions. All RT‐qPCRs were performed with THUNDERBIRD SYBR qPCR Mix (Toyobo Co., Ltd., Kita‐ku, Osaka, Japan) and an MX3000P Real‐Time qPCR System (Stratagene, Santa Clara, California, USA). Primer sets qURA3A_F3 plus qURA3A_R3, qURA3B_F2 plus qURA3B_R2 and 8436qF_ref plus 8436qR_ref were used to detect transcripts of *URA3A*, *URA3B* and *ribosomal protein I5*, respectively. The primer sequences used in the experiments are listed in Table S3. Plasmids with the coding sequences of *URA3A* and *URA3B* were used as standards for the absolute quantification of *URA3A* and *URA3B* transcripts.

### Fungal infection assay of cucumber

Fungal strains were cultured on PDA at 24 °C for 6 days in the dark. Then, their conidia were collected by centrifugation at 3000 ***g*** for 5 min and washed twice with sterilized water. Droplets (10 µL) of conidia at 5 × 10^5^ conidia/mL in water were inoculated onto cucumber cotyledons at 10 days post‐germination. Seeds of *C. sativus, *cucumber Suyo strain (Sakata Seed Corp., Yokohama, Kanagawa, Japan), were planted in a mix of equal amounts of Supermix A (Sakata Seed Corp.) and vermiculite. Then, cucumbers were grown at 24 °C under a 10‐h light/14‐h dark cycle.

### Alignment of URA3 proteins

The amino acid sequence of *Saccharomyces cerevisiae *Ura3p and *C. orbiculare* URA3A and URA3B were aligned using MAFFT software (Katoh *et al.*, 2002). Aligned sequences were formatted using CLC Genomics Workbench8.0 (CLC bio, Aarhus, Midtjylland, Denmark).

## Accession numbers

Ura3p (SGD:S000000747) from the Saccharomyces Genome Database (https://www.yeastgenome.org/). URA3A (ENH84876.1), URA3B (ENH87716.1), PKS1 (ENH81867.1), DMAT3 (ENH86929.1) and *F. oxysporum* f. sp.* cubense *URA3 (EMT68416.1) from the GenBank databases.

## Supporting information


**Fig. S1** ****Amino acid sequence alignment of *Saccharomyces cerevisiae *Ura3p with *Colletotrichum orbiculare *URA3A and URA3B, and *Fusarium oxysporum* URA3.

**Fig. S2** ****The lesser disease symptom phenotype of *ura3a/b* is partially complemented by externally added uridine.

**Fig. S3** *Colletotrichum orbiculare btcAco* knock‐out mutants do not show reduced virulence on cucumber leaves. The experiment was performed in the same conditions as in Fig. 5d.

**Table S1** ****Fungal strain list.

**Table S2** ****Plasmid list.

**Table S3** ****Oligo list.

**Data S1** ****DNA sequences of PCR‐10 and PCR‐11.
