# Supplementary figures and images for: Establishment of a selection marker recycling system for sequential transformation of the plant‐pathogenic fungus Colletotrichum orbiculare
Source: Mol Plant Pathol. 2018 Dec 5;20(3):447–59. doi: 10.1111/mpp.12766 (PMC6637883; doi:10.1111/mpp.12766)

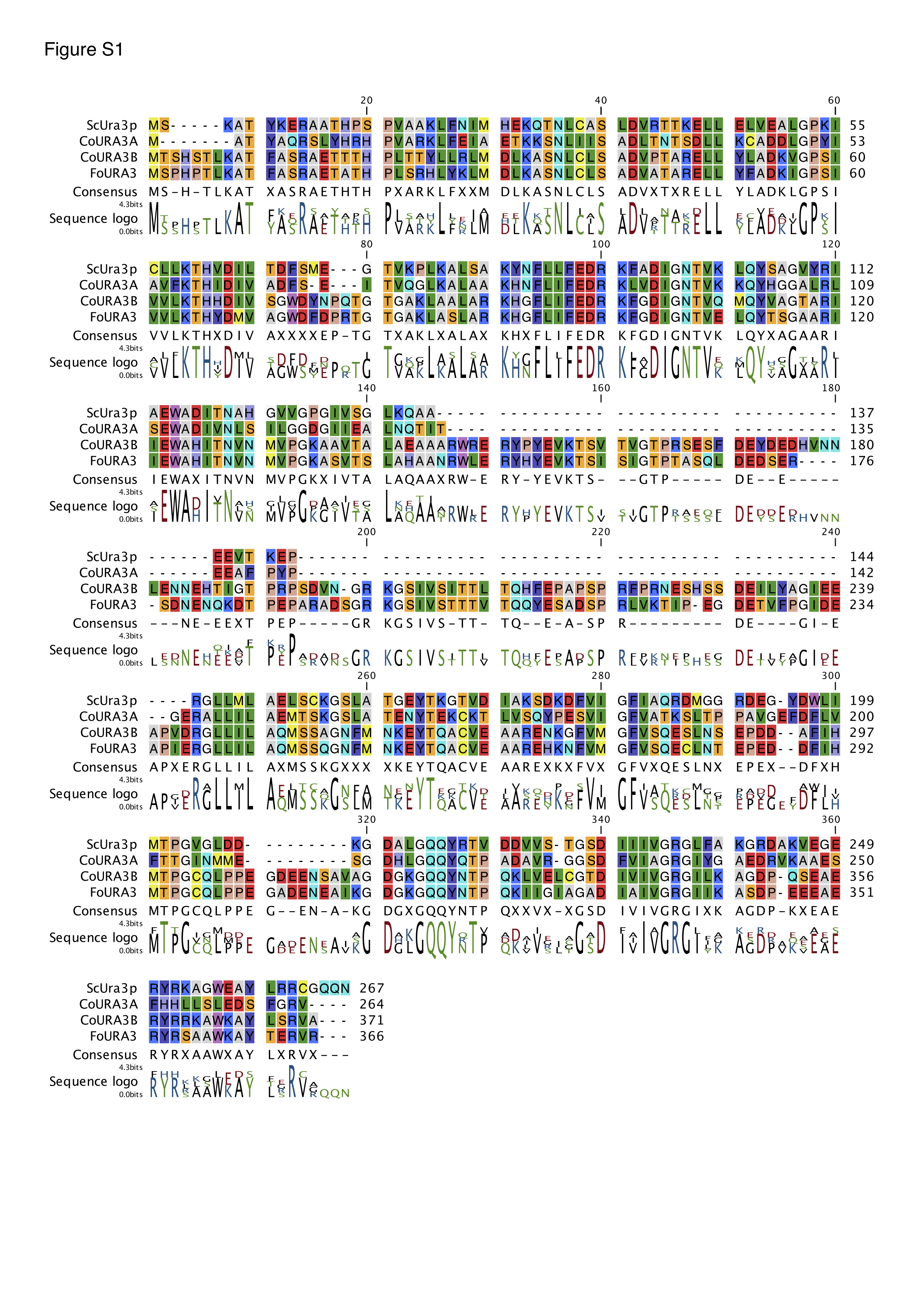

Supplement: Supplementary file 1 — Fig. S1 Amino acid sequence alignment of Saccharomyces cerevisiae Ura3p with Colletotrichum orbiculare URA3A and URA3B, and Fusarium oxysporum URA3. [file MPP-20-447-s001.tif]

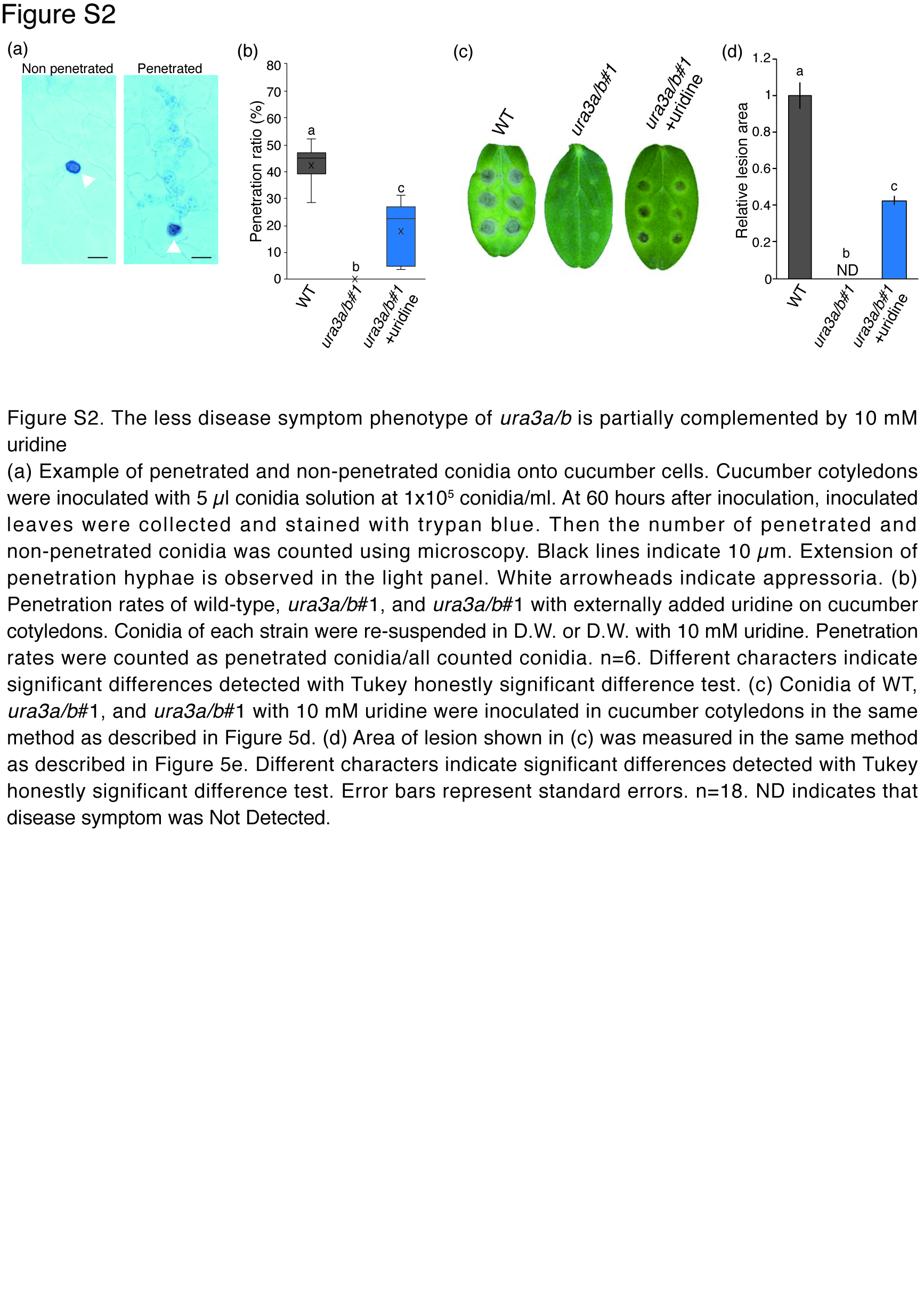

Supplement: Supplementary file 2 — Fig. S2 The lesser disease symptom phenotype of ura3a/b is partially complemented by externally added uridine. [file MPP-20-447-s002.tif]

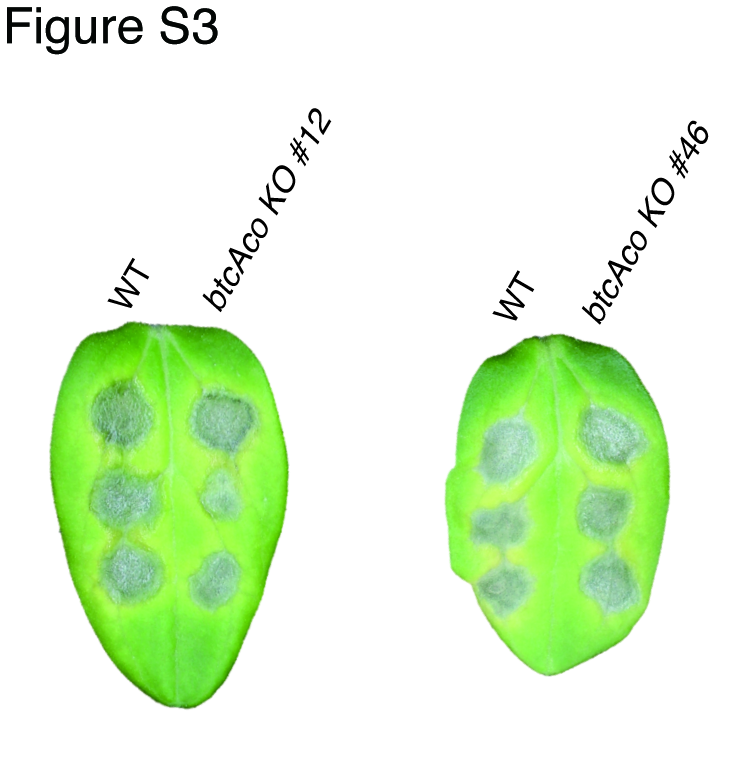

Supplement: Supplementary file 3 — Fig. S3 Colletotrichum orbiculare btcAco knock‐out mutants do not show reduced virulence on cucumber leaves. The experiment was performed in the same conditions as in Fig. 5d. [file MPP-20-447-s003.tif]
