# Supplementary material for: Establishment of a selection marker recycling system for sequential transformation of the plant‐pathogenic fungus Colletotrichum orbiculare
Source: Mol Plant Pathol. 2018 Dec 5;20(3):447–59. doi: 10.1111/mpp.12766 (PMC6637883; doi:10.1111/mpp.12766)
Supplement: Supplementary file 7 — Data S1 DNA sequences of PCR‐10 and PCR‐11. [file MPP-20-447-s007.docx]

Data S1

>PCR-10

tcagtctctaccctcataccccttgGACCCTTTGGCTCGCTTAGTCAGTGCGCCCACTCACTCACACTCAAAAAGGCCACCCCTCCCGCACCCTCTTCTCATCACCGTCTTCATACCACGGTTCGTCAAGCAATCGTATCTGGTAAGCTTTGACCTCCTCGAGCGGGCTCCACTTTGCTATTTCTTGGATCTGCTCTTTCTTTTCTCTCTACCTCTTTTTCTAACCTCTCTTCAGAAAGTTCAACCGTACTTCACTCCATCTTCCATACATCACTCTAGAGGCAGACACAatgacttcacacagcacctt

*The blue colored region is the *Tef* promoter sequence.

>PCR-11

atctctctcgcgtagcgtagGGATCCCCCGGGCTGCAGGAATTCGATATCAAGCTTAGCGGCGTGCTCTGCACATAACACGTGTCGTGTTTGGGTTCGGTATGGGTAATGGCGAATGGGGACATGCATTTATGGGATAGGGGGcgactatacacagtagtccctcctc

*The green colored region is the *SCD1* terminator sequence.
